# Report from the World Health Organization's immunization and vaccines-related implementation research advisory committee (IVIR-AC) ad hoc meeting, 28 June – 1 July 2024

**DOI:** 10.1016/j.vaccine.2024.126307

**Published:** 2024-12-02

**Authors:** Philipp Lambach, Sheetal Silal, Alyssa N. Sbarra, Natasha S. Crowcroft, Kurt Frey, Matt Ferrari, Emilia Vynnycky, C. Jessica E. Metcalf, Amy K. Winter, Laura Zimmerman, Mitsuki Koh, Meru Sheel, Sun-Young Kim, Patrick K. Munywoki, Allison Portnoy, Rakesh Aggarwal, Habib Hasan Farooqui, Stefan Flasche, Alexandra B. Hogan, Kathy Leung, William J. Moss, Xuan-Yi Wang

**Affiliations:** a Immunizations, Vaccines and Biologicals, World Health Organization, Geneva, Switzerland; b Modelling and Simulation Hub, Africa, University of Cape Town, Cape Town, South Africa; c Centre for Global Health, Nuffield Department of Medicine, Oxford University, Oxford, United Kingdom; d Johns Hopkins Bloomberg School of Public Health, Baltimore, United States; e Institute for Disease Modeling, Bill & Melinda Gates Foundation, Seattle, United States; f Pennsylvania State University, State College, United States; g UKHSA, London, United Kingdom; h London School of Hygiene & Tropical Medicine, London, United Kingdom; i Department of Ecology and Evolutionary Biology, Princeton University, Princeton, United States; j Department of Epidemiology and Biostatistics & Center for the Ecology of Infectious Diseases, University of Georgia, Athens, United States; k Centers for Disease Control and Prevention, Atlanta, United States; l School of Public Health, Faculty of Medicine and Health, University of Sydney, Sydney, Australia; m Sydney Infectious Diseases Institute, Faculty of Medicine and Health, University of Sydney, Sydney, Australia; n Seoul National University, Seoul, South Korea; o Kenya Medical Research Institute, Centre for Global Health Research, Nairobi, Kenya; p Department of Global Health, Boston University School of Public Health, Boston, United States; q Center for Health Decision Science, Harvard T.H. Chan School of Public Health, Boston, United States; r Jawaharlal Institute of Postgraduate Medical Education & Research, Puducherry, India; s College of Medicine, QU Health, Qatar University; t Centre for Global Health, Charite, Berlin, Germany; u School of Population Health, University of New South Wales, Sydney, Australia; v School of Public Health, The University of Hong Kong, Hong Kong SAR, China; w Shanghai Institute of Infectious Disease and Biosecurity, Fudan University, Shanghai, China

**Keywords:** Rubella, RCV, Congenital rubella syndrome, IVIR-AC

## Abstract

The World Health Organization's Immunization and Vaccines-related Implementation Research Advisory Committee (IVIR-AC) serves to independently review and evaluate vaccine-related research to maximize the potential impact of vaccination programs. From 28 June – 1 July 2024, IVIR-AC was convened for an ad hoc meeting to discuss new evidence on criteria for rubella vaccine introduction and the risk of congenital rubella syndrome. This report summarizes background information on rubella virus transmission and the burden of congenital rubella syndrome, meeting structure and presentations, proceedings, and recommendations.

## 1 Background

Rubella is an acute viral infection that usually presents with mild symptoms, including fever, rash, and lymphadenopathy, in children and adolescents and is transmitted by respiratory droplets [1]. Rubella virus infection rarely results in severe clinical outcomes (e.g., 1 in 6000 infections develop post-infection encephalitis [2]). However, rubella virus acquired just prior to conception or during pregnancy, particularly in the early weeks of gestation, can lead to fetal death, miscarriage, or congenital rubella syndrome (CRS). Among women infected with rubella virus during the first 8–10 weeks of pregnancy, multiple fetal congenital abnormalities can occur in 90 % of cases [3]. Infants born with CRS can have auditory, ophthalmic, cardiac, and craniofacial anomalies and suffer severe developmental disabilities and delays.

Immunization against rubella is available with a live, attenuated vaccine usually given in conjunction with a measles-containing vaccine (MCV) in the form of measles- and rubella-combination (i.e., MR) or measles-, mumps- and rubella-combination (i.e., MMR). A single dose of any rubella-containing vaccine (RCV) provides long-term protection and has approximately 95 % efficacy. In the pre-vaccine era, rubella virus circulated seasonally and caused large, variable outbreaks occurring annually or every five to nine years. Reported pre-vaccine CRS incidence was 0.1–0.2 per 1000 live births, rising to 0.8–4 per 1000 live births during epidemics [[4], [5], [6], [7]]. The estimated CRS incidence in 2019 was 64 (95 % confidence interval (CI): 24–123) per 100,000 live births in the African Region and 27 (95 % CI: 4–67) per 100,000 live births in the Eastern Mediterranean Region [8]. In regions where most countries have introduced RCV, CRS incidence in 2019 is estimated to be low (i.e., less than 1 per 100,000 live births (95 % CI: less than 1–8) in the South-East Asian Region and less than 1 per 100,000 live births (95 % CI: less than 1–12) in the Western Pacific Region; similar estimates are found in the European Region and in the Region of the Americas. Through immunization, rubella elimination has been achieved in more than 50 % of all countries and deemed feasible to achieve in others [9].

### 1.1 Paradoxical effect of RCV introduction

Countries with the highest incidence of CRS are those without RCV in their immunization programs. In the absence of vaccine, most women were immune to rubella prior to childbearing age. However, susceptibility among women of childbearing age can increase if a wide age range introduction campaign is not conducted, routine RCV coverage is low, and regular follow-up campaigns are absent. This increase in susceptibility is due to an increasing average age at time of infection, and for rubella may lead to increases in CRS incidence. This phenomenon has been referred to as the “paradoxical effect”; it has been demonstrated theoretically using mathematical models [10,11] and may have been observed in Costa Rica [12] and Greece [13], where there were no wide age range introductory campaigns, low routine RCV coverage, and no follow-up campaigns. However, reducing rubella transmission through increasing vaccination coverage was sufficient to prevent further CRS cases in these countries. The paradoxical effect can be avoided if coverage levels are sufficiently high, which have been historically estimated to be approximately 80 %.

The coverage level needed to preempt the paradoxical effect is closely related to the level of disease transmission. As birth rates in many countries decrease with demographic transitions, transmission of rubella generally decreases and sufficient vaccination coverage levels to prevent an increase in CRS burden are estimated to be reduced [11]. Similarly, a lower R_0_, the basic reproduction number (i.e., the number of secondary infectious people resulting from a single infectious individual in a completely susceptible population), results in a lower sufficient rubella vaccination coverage to prevent increases in CRS burden. Historically, the R_0_ for rubella has been estimated to vary from 2.4 to 7.8 in Europe [14,15] and 6.9 to 11.8 in Addis Ababa, Ethiopia [16]. As a result, early modeling exercises to assess the impact of rubella vaccination would assume equally likely R_0_ values between 5 and 12 [11], [17]. Recent estimates of R_0_, however, are lower than those previously estimated. In a recent global assessment [18], R_0_ was estimated in African, Eastern Mediterranean, Western Pacific, and South East Asian regions to be less than five in most settings and typically ranging between 2 and 3. The typical range for R_0_ in the Americas was 3–4 and 4–7 in the European region.

### 1.2 Current recommendation for RCV introduction

The World Health Organization (WHO) recommends the introduction of RCV into national immunization programs to reduce the burden of CRS and ultimately eliminate rubella [19]. With MR combination vaccines, WHO recognizes that progress on rubella vaccination and elimination will also reduce measles burden, particularly via the recommended wide age range catch-up campaigns at the time of RCV introduction, and accelerate progress towards measles elimination. Measles vaccination programs can serve as a platform for RCV introduction. In 2000, WHO recommended RCV introduction for countries that can achieve 80 % or greater coverage, as evidenced by achieving at least 80 % first-dose coverage of any measles-containing vaccine (MCV1) through routine immunization programs. However, WHO recommends careful consideration prior to introducing RCV to ensure a long-term commitment to sustaining sufficiently high vaccination coverage.

In 2010, the Strategic Advisory Group of Experts on Immunization (SAGE) recommended that the 80 % criterion should be extended to consider campaign coverage of any MCV. SAGE also recommended that, in addition to meeting an 80 % coverage threshold, countries should introduce RCV alongside a wide age range catch-up campaign (e.g., targeting children aged 9 months to 15 years, or based on the susceptibility profile across birth cohorts). Immediately following this initial catch-up campaign, RCV should be introduced into the routine immunization program at either 9 or 12 months, depending on the current MCV1 schedule. All follow-up campaigns and measles or rubella outbreak response should be conducted with combination RCV (e.g., MR) rather than monovalent measles vaccines, with campaign target age ranges dependent on country-specific measles and rubella epidemiology. In 2020, SAGE reconfirmed their 2010 recommendations.

Evidence from recent modeling analyses of data from Nigeria [20], a country that has yet to introduce RCV, and generalized transmission and demographic settings across sub-Saharan Africa [21] provide new insights on the potential impact of RCV introduction on increasing CRS burden. In light of demographic changes (i.e., population size and birth rates) and updated estimates of R_0_ consistent with other recent findings [18], these new analyses suggest that reconsideration of guidance for RCV introduction is warranted.

## 2 Scope and objective of meeting

As an advisory board to the WHO Immunization, Vaccines, and Biologicals (IVB) Department and SAGE, the Immunization and Vaccines-related Implementation Research Advisory Committee (IVIR-AC) provides an independent assessment of methodology used across vaccination impact and effectiveness, implementation science, and modeling analyses. IVIR-AC has no executive or decision-making power [22]. As rubella vaccine introduction policy will be discussed during the upcoming SAGE meeting (24–26th September 2024), IVIR-AC was requested to review two new modeling analyses in an ad hoc session outside their bi-annual meeting calendar. Specifically, IVIR-AC was requested to review analyses that explore the risk of the paradoxical effect of RCV introduction on increasing CRS burden and comment on the methodologies. Additionally, IVIR-AC was asked to provide suggestions for any modifications or clarifications that are required and feedback on the plans for future related work.

This report summarizes proceedings and recommendations of the IVIR-AC's virtual ad hoc meeting held from 28th June–1st July 2024 [23]. An overview of each presentation given during the meeting session, committee feedback, and recommendations are described below.

## 3 Description of session

A modeling team from the Institute for Disease Modeling (IDM) presented on findings from a generalized modeling analysis across settings in sub-Saharan Africa [21]. The study team implemented a stochastic, age-specific, agent-based model using Epidemiological MODeling software [24] (EMOD) software that assumed an R_0_ of 5, age-specific susceptibility, and no age-structured infectivity for those who were infectious. Rubella and CRS burden were calculated based on estimates of age-structured incidence (from modeled output) and age-structured fertility rates from the United Nations (UN) World Population Prospects (WPP). In the absence of vaccination (i.e., a scenario when routine immunization coverage equals 0 %), rubella dynamics were estimated to be at equilibrium. With RCV introduction at various routine coverage levels (i.e., 50 %, 60 %, 70 %, and 80 %) and in the absence of a wide age range catch-up campaign, infections were estimated to equilibrate after approximately five years after an initial decrease in rubella cases. However, the age distribution of rubella cases was estimated to continue evolving over 20 years as the demographic profile of populations likewise continued to change. In the scenario with 60 % routine RCV coverage and no catch-up campaign, similar levels of CRS resurgence were estimated compared to what was observed after RCV introduction via routine immunization systems in Costa Rica [12] and Greece [13]. Annual estimates overall suggested yearly variability in transmission, including evidence of both interruption, re-introduction, and outbreak.

The team also presented scenarios with dynamic demographics (including overall population size and declining mortality and fertility rates). When using dynamic populations under the no-vaccination scenario, estimated rubella incidence declined overall, but estimated CRS incidence increased by over 33 % in the next 25 to 30 years. Across routine vaccination scenarios, rubella incidence also was estimated to decline before reaching equilibrium and estimated CRS incidence immediately declined before leading to resurgence approximately 15 years later. These dynamics were different from those observed in previous modeling scenarios as there was a significant reduction in future CRS at levels of coverage (i.e., 60 %) that were associated with an increase in CRS in models that did not account for the demographic changes. However, quantitatively, RCV introduction without an initial catch-up or any follow-up campaigns, which is not recommended, did not increase CRS burden above baseline 15 years later in any scenario apart from an assumption of 50 % coverage. The team has previously applied this model to the Democratic Republic of the Congo and found similar results [25]. Based on their modeled results, the team concluded that overall:

• RCV introduction will lead to immediate declines in CRS;
• in scenarios with no vaccination, there will be increased CRS burden as fertility rates decline;
• incomplete vaccination may lead to CRS resurgence following 15 years post introduction; and
• implementing a wide age range catch-up campaigns at time of introduction and periodic follow-up campaigns will reduce susceptibility accumulations and outbreak risk.

Additionally, a modeling team from Pennsylvania State University (PSU) presented on results from a subnational modeling analysis of CRS risk following RCV introduction in Nigeria [20]. The study team fitted an age-specific catalytic model to state-level serological data from approximately 40,000 children and women collected during the 2018 Nigeria AIDS Indicator and Impact Survey [26]. Using these results, the team then fitted a separate force of infection model assuming age-structured mixing across three age categories (i.e., 0-to-2-years, 3-to-14 years, and 15 years and older). R_0_ was estimated using a next generation matrix approach and found to range between 2.5 and 6.5 with lower values in southern states and higher values in northern states. Nationally, R_0_ was estimated to be between 3.3 and 4.1; both of which were lower than previous estimates of R_0_. These updated R_0_ values were used to parameterize a deterministic, age-structured transmission model of rubella and was calibrated to empirical data from Nigeria. This model assumed that routine RCV coverage varied subnationally and was equal to current MCV1 coverage (i.e., MCV is replaced with an MR combination vaccine). Additionally, the model generated uncertainty from the posterior distribution of estimates of age-specific force of infection.

Overall, the team estimated that catch-up and follow-up campaigns consistent with the expectation of continued measles control efforts would avoid CRS increases in all states. In analyses that only considered routine vaccination (i.e., no catch-up and no follow-up campaigns), northern states, with higher R_0_ and lower MCV1 coverage, were estimated to experience an initial decrease in CRS burden and then an increase in CRS after 10 or more years. Southern states, with lower R_0_ and higher MCV1 coverage, were estimated to experience a decrease in CRS incidence following introduction that persisted across the modeled 30-year period. When aggregated nationally, these results suggested an overall net reduction in annual CRS burden. The team also simulated scenarios of routine coverage and campaigns; in all scenarios, there was a large short-term reduction of CRS incidence with or without catch-up campaigns. The cumulative benefit of RCV introduction on CRS burden varied across states and across vaccination scenarios. The team computed that the minimally sufficient coverage to avoid increased CRS burden is less than 80 % in all states (range between 0 % and 76 %). Among 13 states projected to have an increase in CRS burden at current MCV1 coverage levels, annual increases in MCV1 coverage between 1 and 5 % over 10 to 15 years were estimated to prevent an increase of CRS cases. Using their modeled results, the study team overall concluded that in Nigeria:

• current thresholds of 80 % coverage are overly conservative relative to current demography and rubella epidemiology;
• current routine MCV1 coverage is sufficient to achieve net reduction in CRS cases, and catch-up and follow-up campaigns can prevent CRS increases even at current levels across all states; and
• RCV introduction in Nigeria with wide age range catch up campaigns will result in 11,000 CRS cases averted over 5 years.

To complement the research already performed, a team from PSU also presented on behalf of a group of rubella modelers (University of Georgia (UGA), UK Health Security Agency (UKHSA), IDM) on a proposal for a simulation study to evaluate CRS risk following vaccine introduction in the 19 countries that have yet to introduce RCV into their national immunization program. Building on the team's previous work to understand the feasibility of measles and rubella elimination through modeling various vaccination scenarios [9], the team proposed to project national rubella and CRS burden over a 40-year period following RCV introduction with projected demographic changes in population size and birth rates. The team proposed to apply two separate models of rubella virus transmission currently used for rubella estimation for the Vaccine Impact Modeling Consortium (VIMC). For parameterizing both models, IVIR-AC welcomes the sampling of R_0_ from posterior distributions and recommends prioritizing the use of sub-national serosurveillance data to estimate R_0_ where such data are available. IVIR-AC emphasizes the need to present detailed methodology and assumptions for countries and locations where no serological data are available and alternative estimates for R_0_ are applied.

Fourteen scenarios were proposed for projection simulations: one scenario with no vaccination, one scenario assuming current MCV1 coverage (i.e., coverage does not increase) and no catch-up or follow-up campaigns, four scenarios with current MCV1 coverage and varying catch-up campaign coverage (i.e., 60 %, 70 %, 80 %, 90 %) and no follow-up campaigns, and eight scenarios with current MCV1 coverage with varying catch-up campaign coverage (i.e., 60 %, 70 %, 80 %, 90 %) and varying follow-up campaign coverage (i.e., 60 %, 90 %). Catch-up campaigns are defined as those targeting children aged 6 months to 14 years once at the time of the RCV introduction and follow-up campaigns are defined as those targeting children aged 9 months to 5 years and occurring every 3–4 years to fill gaps in routine immunization coverage. IVIR-AC welcomes the broad set of scenarios that have been proposed for planned simulations of RCV introductions across the 19 countries without RCV. For the scenarios modeling routine immunization only, IVIR-AC encourages consideration of scale-up in coverage to reach operational targets as that will have an impact on CRS burden. If modeled estimates in the current scenario with static routine immunization coverage alone result in projected increases in CRS, IVIR-AC recommends identifying the minimally sufficient coverage threshold to determine the needed improvements to routine coverage to avoid CRS increases without supplementary vaccination campaigns per the Nigeria analysis [20].

The study team proposed to produce outputs of annual and cumulative rubella incidence, annual and cumulative CRS incidence, the proportion of years with a rubella outbreak (defined as 5 infections per 100,000 population), and the effective reproductive number (R_E_) in each year. Results across both models (UGA and UKHSA) will be compared to each other as well as to the published results from the IDM and PSU models presented during the session. Comparisons will be made across metrics of rubella and CRS burden as well as risk of exceeding baseline CRS incidence and time to exceeding CRS incidence. Additionally, the team proposed exploring comparisons of results from national-level models to results from subnational data. When comparing subnational and national models in the Democratic Republic of the Congo and Nigeria, IVIR-AC recommends a clear reporting of projected differences and the associated implications for the national-level model projections in the remaining 17 countries. While subnational modeling may be ideal to capture relevant heterogeneity, IVIR-AC recommends that outputs from the proposed national-level models, which are likely to present more optimistic projections for CRS burden, are appropriately contextualized in each of the 19 countries. Given the heterogeneity in MCV coverage and implementation challenges (e.g., access to health services, ongoing conflict) in many of the 19 countries, the results should be caveated with suggestions of potential sub-national impact on coverage and CRS burden.

Across the 19 countries, IVIR-AC recommends conducting similar threshold-based analyses to estimate minimally sufficient levels of both catch-up and follow-up campaign coverage necessary to prevent increase in CRS burden. IVIR-AC recommends additional scenario modeling for catch-up campaigns introducing age-based coverage thresholds for 9–59 months and 5–14 years to account for possible reduced coverage among older children. Wherever possible, IVIR-AC recommends the use of historical coverage data from measles supplementary immunization activities to inform coverage thresholds for the proposed scenario modeling. Where data are not available, assumptions around coverage threshold should be presented as rationale.

Overall, IVIR-AC finds the modeling analyses of rubella virus transmission presented by teams from IDM and PSU to be sound and supports the proposed use of VIMC models to simulate RCV introduction in the 19 countries that have not yet introduced. Due to the changes in population demographics, IVIR-AC recognizes an increasing risk of CRS even in the absence of rubella vaccination. RCV introduction can interrupt transmission of rubella virus and reduce the projected future burden of CRS, and thus promote the elimination of rubella. As Gavi, the Vaccine Alliance, follows current WHO recommended policy and most remaining countries are Gavi-eligible, it is expected that RCV introduction would be implemented using standard introductory catch-up campaigns and later follow-up campaigns. However, IVIR-AC recognizes the continuing risk of observing the paradoxical effect (i.e., increasing CRS in the presence of vaccination) resulting from suboptimal coverage following RCV introduction if the recommended catch-up campaign at introduction and subsequent follow-up campaigns are not implemented. IVIR-AC acknowledges that the modeled increase is not anticipated to be immediate but over a period of time, beginning approximately 10 years from vaccine introduction as susceptible populations accumulate if RCV coverage were to remain suboptimal. IVIR-AC strongly supports the proposed modeling with multiple scenarios and outcomes being considered for RCV introduction, including various combinations of coverage achieved through routine immunization and both catch-up and follow-up campaigns. Across all modeling analyses, IVIR-AC recommends non-specialist communication of the different modeling scenarios being applied for RCV introduction. The modeling results should be presented in the context of future implementation. Additionally, IVIR-AC recommends countries develop a risk communication strategy to mitigate any adverse impact on vaccine confidence if outbreaks of rubella or CRS were experienced 15–20 years after a rubella vaccination program was introduced.

## 4 Conclusions

New available modeling evidence suggests that demographic changes can result in increased CRS incidence in the absence of vaccination. However, this increase in CRS cases may be mitigated by carefully planned RCV introduction and sustained coverage at sufficient levels. After reviewing the presented methods and evidence, IVIR-AC:

• finds that the methodology used to address these questions is appropriate;
• welcomes the additional projection modeling analyses and recommends supplementary scenarios to complement those planned; and
• emphasizes the need for transparent, nuanced, and contextualized communication of methodologic assumptions and scenario results.

SAGE will review the evidence presented during this meeting, and the corresponding IVIR-AC recommendations and feedback, during their upcoming September meeting. IVIR-AC will next convene for their biannual meeting from 9 to 13 September 2024.

## WHO author disclaimer

P. L., N. S. C. and M. K. work for the World Health Organization (WHO). The authors alone are responsible for the views expressed in this publication and they do not necessarily represent the decisions, policy or views of the WHO.

## CRediT authorship contribution statement

**Philipp Lambach:** Conceptualization, Project administration, Supervision, Writing – review & editing. **Sheetal Silal:** Investigation, Supervision, Writing – review & editing. **Alyssa N. Sbarra:** Conceptualization, Writing – original draft. **Natasha S. Crowcroft:** Supervision, Writing – review & editing. **Kurt Frey:** Investigation, Writing – review & editing. **Matt Ferrari:** Investigation, Writing – review & editing. **Emilia Vynnycky:** Writing – review & editing. **C. Jessica E. Metcalf:** Writing – review & editing. **Amy K. Winter:** Writing – review & editing. **Laura Zimmerman:** Writing – review & editing. **Mitsuki Koh:** Project administration, Writing – review & editing. **Meru Sheel:** Investigation, Writing – review & editing. **Sun-Young Kim:** Investigation, Writing – review & editing. **Patrick K. Munywoki:** Investigation, Writing – review & editing. **Allison Portnoy:** Investigation, Writing – review & editing. **Rakesh Aggarwal:** Investigation, Writing – review & editing. **Habib Hasan Farooqui:** Investigation, Writing – review & editing. **Stefan Flasche:** Investigation, Writing – review & editing. **Alexandra B. Hogan:** Investigation, Writing – review & editing. **Kathy Leung:** Investigation, Writing – review & editing. **William J. Moss:** Investigation, Writing – review & editing. **Xuan-Yi Wang:** Investigation, Writing – review & editing.

## Declaration of competing interest

P. L. was supported by the 10.13039/100000865Bill & Melinda Gates Foundation for this work. S. S. was supported by the 10.13039/100004423World Health Organization for this work. A. N. S. was financially supported by the 10.13039/100004423World Health Organization for this work, and is additionally supported by the 10.13039/100000865Bill & Melinda Gates Foundation, Gavi, the Vaccine Alliance, and the 10.13039/100000002National Institutes of Health. K. F. is employed by the Bill & Melinda Gates Foundation. M. F. is supported by the 10.13039/100000001National Science Foundation, the 10.13039/100000865Bill & Melinda Gates Foundation, Gavi, the Vaccine Alliance, 10.13039/100000030Centers for Disease Control and Prevention, and 10.13039/501100000761Imperial College London. E. V. was supported for this work by Gavi, the Vaccine Alliance via the Vaccine Impact Modeling Consortium (VIMC), which is jointly funded by Gavi, the Vaccine Alliance and the Bill & Melinda Gates Foundation. C. J. E. M. has previously received travel funds to visit the Max Plank Institute of Evolutionary Anthropology. A. K. W. was supported for this work by Gavi, the Vaccine Alliance, is additionally supported by the 10.13039/100000865Bill & Melinda Gates Foundation, and has previously received travel funds from Gavi, the Vaccine Alliance. M. K. was supported for this work by the Bill & Melinda Gates Foundation. A. P. is supported by 10.13039/100001125Gavi, the Vaccine Alliance, 10.13039/501100000761Imperial College London, the 10.13039/100000865Bill & Melinda Gates Foundation, and the 10.13039/100004423World Health Organization. A. B. H. was supported by the Australian 10.13039/501100000925National Health and Medical Research Council for this work, is additionally supported by the Australian 10.13039/501100009287NSW Ministry of Health, PATH, the 10.13039/100004423World Health Organization, and Gavi, the Vaccine Alliance, and has received consulting fees from the Australian 10.13039/501100009287NSW Ministry of Health, WHO Europe and 10.13039/100004425Asian Development Bank. A. N. S. and M. F. report travel related support from the 10.13039/100004423World Health Organization to attend previous IVIR-AC meetings. All other authors have no declarations.

## Data Availability

There is no data contained within this report. For additional details, please see: https://www.who.int/news-room/events/detail/2024/06/28/default-calendar/immunization-and-vaccines-related-implementation-research-advisory-committee-(ivir-ac)---ad-hoc-june-2024.

## References

[1] Winter A.K., Moss W.J. (2022). Rubella. Lancet.

[2] Pinkbook (2022). Rubella | CDC. https://www.cdc.gov/vaccines/pubs/pinkbook/rubella.html.

[3] Miller E., Cradock-Watson John E., Pollock Thomas M. (1982). Consequences of confirmed maternal rubella at successive stages of pregnancy. Lancet.

[4] Cutts F.T., Robertson S.E., Diaz-Ortega J.L., Samuel R. (1997). Control of rubella and congenital rubella syndrome (CRS) in developing countries, part 1: burden of disease from CRS. Bull World Health Organ.

[5] Lawn J.E. (2000). Unseen blindness, unheard deafness, and unrecorded death and disability: congenital rubella in Kumasi. Ghana Am J Public Health.

[6] Enders G., Miller E., Nickerl-Pacher U., Cradock-Watson J.E. (1989). Outcome of Confirmed Periconceptional Maternal rubella. Obstet Gynecol Surv.

[7] Thant K.-Z. (2006). Active surveillance for congenital rubella syndrome in Yangon. Myanmar *Bull World Health Organ*.

[8] Vynnycky E. (2023). Estimates of the global burden of congenital rubella syndrome, 1996-2019. Int J Infect Dis.

[9] Winter A.K. (2022). Feasibility of measles and rubella vaccination programmes for disease elimination: a modelling study. Lancet Glob Health.

[10] Knox E.G. (1980). Strategy for rubella vaccination. Int J Epidemiol.

[11] Metcalf C.J.E., Lessler J., Klepac P., Cutts F., Grenfell B.T. (2012). Impact of birth rate, seasonality and transmission rate on minimum levels of coverage needed for rubella vaccination. Epidemiol Infect.

[12] Jiménez G. (2007). Estimating the burden of congenital rubella syndrome in Costa Rica, 1996–2001. Pediatr Infect Dis J.

[13] Panagiotopoulos T., Georgakopoulou T. (2004). Epidemiology of rubella and congenital rubella syndrome in Greece, 1994-2003. Eurosurveillance.

[14] Edmunds W.J. (2000). The pre-vaccination epidemiology of measles, mumps and rubella in Europe: implications for modelling studies. Epidemiol Infect.

[15] https://rss.onlinelibrary.wiley.com/doi/abs/10.1111/1467-9876.00233.

[16] Cutts F.T. (2000). Sero-epidemiology of rubella in the urban population of Addis Ababa. Ethiopia Epidemiol Infect.

[17] Vynnycky E., Gay N.J., Cutts F.T. (2003). The predicted impact of private sector MMR vaccination on the burden of congenital rubella syndrome. Vaccine.

[18] Papadopoulos T., Vynnycky E. (2022). Estimates of the basic reproduction number for rubella using seroprevalence data and indicator-based approaches. PLoS Comput Biol.

[19] World Health Organization (2020). Rubella vaccines: WHO position paper – July 2020. Wkly Epidemiol Rec.

[20] Nakase T. (2024). The impact of sub-national heterogeneities in demography and epidemiology on the introduction of rubella vaccination programs in Nigeria. Vaccine.

[21] Frey K. (2024). Congenital rubella syndrome does not increase with introduction of rubella-containing vaccine. Vaccines.

[22] https://www.who.int/publications/m/item/terms-of-reference-for-the-immunization-and-vaccines-related-implementation-research-advisory-committee-(ivir-ac).

[23] https://www.who.int/news-room/events/detail/2024/06/28/default-calendar/immunization-and-vaccines-related-implementation-research-advisory-committee-(ivir-ac)---ad-hoc-june-2024.

[24] Institute for Disease Modeling (2024). Welcome to EMOD modeling for general disease. https://docs.idmod.org/projects/emod-generic/en/2.20_a/.

[25] Cheng A. (2021). Examination of scenarios introducing rubella vaccine in the Democratic Republic of the Congo. Vaccine X.

[26] https://naiis.ng/.

